# Pharmacologic profile of ITI-333: a novel molecule for treatment of substance use disorders

**DOI:** 10.1007/s00213-024-06578-w

**Published:** 2024-05-06

**Authors:** Gretchen L. Snyder, Peng Li, Terry Martin, Lei Zhang, Wei Yao, Hailin Zheng, David R. Maguire, Lisa R. Gerak, Kimberly E. Vanover, Charles P. France, Robert Davis

**Affiliations:** 1 Intra-Cellular Therapies Inc., 430 East 29th Street, Suite 900, New York, NY 10016 USA; 2 University of Texas Health Science Center at San Antonio, 7703 Floyd Curl Drive (Mail Code 7764), San Antonio, TX 78229-3900 USA; 3 Addiction Research, Treatment and Training Center of Excellence, University of Texas Health Science Center at San Antonio, San Antonio, TX USA; 4 Engrail Therapeutics, San Diego, CA 92130 USA

**Keywords:** 5-HT_2A_ receptor, Mu opioid receptor, Partial agonist, Beta arrestin, Opioid use disorder, Morphine, Cue-induced heroin reinstatement

## Abstract

**Rationale:**

Medications are urgently needed to treat symptoms of drug withdrawal and mitigate dysphoria and psychiatric comorbidities that drive opioid abuse and relapse. ITI-333 is a novel molecule in development for treatment of substance use disorders, psychiatric comorbidities, and pain.

**Objective:**

Characterize the preclinical profile of ITI-333 using pharmacological, behavioral, and physiological assays.

**Methods:**

Cell-based assays were used to measure receptor binding and intrinsic efficacy of ITI-333; animal models were employed to assess effects on opioid reinstatement, precipitated oxycodone withdrawal, and drug abuse liability.

**Results:**

In vitro, ITI-333 is a potent 5-HT_2A_ receptor antagonist (K_i_ = 8 nM) and a biased, partial agonist at μ-opioid (MOP) receptors (K_i_ = 11 nM; lacking β-arrestin agonism) with lesser antagonist activity at adrenergic α_1A_ (K_i_ = 28 nM) and dopamine D_1_ (K_i_ = 50 nM) receptors. In vivo, ITI-333 blocks 5-HT_2A_ receptor-mediated head twitch and MOP receptor-mediated effects on motor hyperactivity in mice. ITI-333 alone is a naloxone-sensitive analgesic (mice) which suppresses somatic signs of naloxone-precipitated oxycodone withdrawal (mice) and heroin cue-induced reinstatement responding without apparent tolerance or physical dependence after chronic dosing (rats). ITI-333 did not acutely impair gastrointestinal or pulmonary function (rats) and was not intravenously self-administered by heroin-maintained rats or rhesus monkeys.

**Conclusions:**

ITI-333 acts as a potent 5-HT_2A_ receptor antagonist, as well a biased MOP receptor partial agonist with low intrinsic efficacy. ITI-333 mitigates opioid withdrawal/reinstatement, supporting its potential utility as a treatment for OUD.

**Supplementary Information:**

The online version contains supplementary material available at 10.1007/s00213-024-06578-w.

## Introduction

Overdoses from opioids, including prescription pain medications, have dramatically increased over the past decade in the United States (Scholl et al. 2019). It is estimated that of the 70,630 drug overdose deaths in the USA in 2019, over 70% involved an opioid (US Centers for Disease Control and Prevention 2021). Further, overdose deaths in the US are in large part related to increased availability of prescription pain medications, heroin, and, more recently, synthetic opiates (e.g. fentanyl). The total economic burden of opioid use disorder (OUD) and fatal overdoses in the United States was estimated at $1.02 trillion in 2017 alone (Florence et al. 2021). This public health crisis is being addressed by a search for new and innovative medications to treat OUD.

Prescription pain medications with abuse-deterrent claims are now entering the market, along with medication combinations (e.g., Suboxone®, other buprenorphine/naloxone combinations including long acting products, lofexidine, opioid dependence vaccines) that aid in alleviating some of the physical symptoms of opioid withdrawal (Kampman and Jarvis 2015; Schuckit 2016; McCarty et al. 2018). However, these medications may require full detoxification from opioids prior to use, which can induce dysphoric physical and mood disturbances that drive psychiatric comorbidities (e.g., anxiety, depression) and trigger reinstatement of drug taking and relapse to opioid abuse. As a result, as many as 80% of individuals who terminate opioid use relapse within 2 years (Bart 2012).

New medications capable of more effectively treating OUD are thus urgently needed. These include medications that lack abuse liability and other safety concerns commonly associated with opioid use (e.g., see Dufort & Samaan 2021 and Florence et al. 2021). Moreover, these medications would ideally ease the uncomfortable physical symptoms of opioid withdrawal without requiring full detoxification (like naltrexone) before use. Furthermore, medications capable of mitigating the comorbid psychiatric symptoms and mood disturbances that drive opioid use and precipitate relapse are currently lacking and would be highly valuable. In addition, medications that can alleviate pain without eliciting physical dependence and tolerance are needed and may be a key component in preventing the recurrent cycle of licit/illicit opioid use that often results in OUD.

Here, we describe the in vitro and in vivo pharmacology of ITI-333, a novel, brain-penetrant, orally bioavailable compound that acts as a potent antagonist (low nM K_i_) at 5-HT_2A_ receptors and partial, biased agonist at MOP receptors. ITI-333 also displays lower affinity antagonism at D_1_ and adrenergic α_1A_ receptors. In cell-based and in vivo animal assays, ITI-333 displays properties anticipated to aid in opioid withdrawal and prevent opioid relapse for sustainable long-term recovery. Additionally, ITI-333 has a low liability for abuse and safety issues in self-administration, physical tolerance/dependence, and gastrointestinal (GI) and pulmonary function assays.

## Materials and Methods

### Materials

**Fig. 1:**
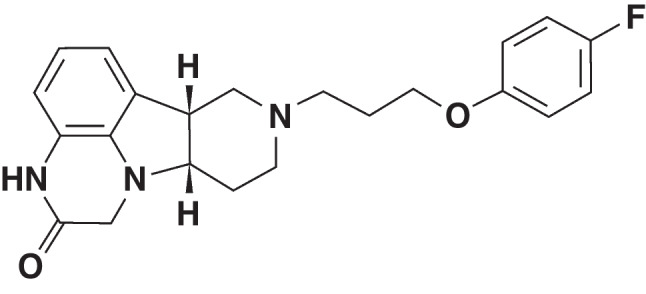
Structure of ITI-333**.** ITI-333, or (6b*R*,10a*S*)-8-[3-(4-fluorophenoxy)propyl]-6b,7,8,9,10,10a-hexahydro-1*H*-pyrido[3’,4’:4,5]pyrrolo[1,2,3-*de*]quinoxaline-2(3*H*)-one (MW = 381.4 g/mol)

All reagents used for these studies were of the highest purity available and were obtained from Sigma-Aldrich (St Louis, MO) unless otherwise stated. Heroin hydrochloride was provided by the Drug Supply Program of the National Institute on Drug Abuse, Rockville, MD) unless otherwise indicated. Oxycodone was obtained from Mallinkrodt Pharmaceuticals (Dublin, Ireland). ITI-333, or (6b*R*,10a*S*)-8-[3-(4-fluorophenoxy)propyl]-6b,7,8,9,10,10a-hexahydro-1H-pyrido[3’,4’:4,5]pyrrolo[1,2,3-de]quinoxaline-2(3H)-one (Fig. 1), a novel molecule discovered and synthesized in the Medicinal Chemistry Department at ITCI (New York, NY), was administered in all studies as a free base (MW = 381.44 g/mol). Unless otherwise stated, ITI-333 was prepared for in vivo subcutaneous (s.c.) dosing in a vehicle composed of 45% Trappsol (in water) and administered in volumes of 6.67–10 mL/kg.

### Animals

All animals were cared for in accordance with the the *Guide for the Care and Use of Laboratory Animals* of the Institute of Laboratory Animal Resources, National Research Council, and all procedures were performed with the approval of the Institutional Animal Care and Use Committees at the respective institutions and contract research organizations. All rodents were experimentally naïve at the start of experiments and were tested only once, unless otherwise stated. All monkeys had received opioids in previous studies but were drug-free for at least two weeks prior to this study. Animal numbers and treatment group sizes for all experiments described here were based on prior studies in the individual laboratories or contract research organizations conducting the work. The minimal number of animals sufficient to obtain statistically reliable results was used.

### Receptor binding assays

The binding of ITI-333 to principal receptors of interest was measured in cell-based assays (Eurofins, Celle l’Evescault, France). A broad selectivity screen of 44 neurotransmitter receptors, enzymes, and channels was first used to test for binding of ITI-333 (0.1 µM) to a diverse panel of potential off-target proteins. Binding affinity was measured and expressed as a % inhibition of control-specific binding; inhibition of greater than 50% was considered to represent significant effects of ITI-333 on a given target. Receptor targets demonstrating > 50% binding with ITI-333 in the broad target screen—including 5-HT_2A_, MOP, dopamine D_1_ and D_2_, and adrenergic α_1A_ receptors—were investigated in further detail in individual assays for binding affinity and for functional activity using recombinant human receptors expressed in Chinese Hamster Ovary (CHO) cells and standard methods. The affinity of ITI-333 was determined at 5-HT_2A_, MOP, D_1_, D_2_, and α_1A_ receptors by measuring inhibition of binding of 0.1 nM [^125^I]DOI, 0.35 nM [^3^H]DAMGO, 0.3 nM [^3^H]SCH23390, 0.3 nM [^3^H]methylspiperone, and 0.1 nM [^3^H]prazosin, respectively.

### In vitro functional assays

#### 5-HT_2A_ receptors

ITI-333 functional activity was evaluated in a whole cell-based assay system in which 5-HT_2A_ receptor-dependent calcium signaling was measured using a recombinant calcium-dependent bioluminescent protein. ITI-333 was studied in the agonist mode and for its ability to antagonize the activity of the 5-HT_2A_ receptor full agonist α-methylserotonin.

#### MOP receptors

ITI-333 functional activity was examined in CHO cells expressing human recombinant MOP receptors; agonist activity (using 1 µM DAMGO as a control) and antagonist activity (using reversal of DAMGO-inhibited cAMP production) were assessed at drug concentrations between 0.056 nM–10 µM. Buprenorphine and naloxone (0.0056 nM–1 μM) were used as comparators for agonist and antagonist activity, respectively. The functional activity of ITI-333 at MOP receptors was further investigated in a whole cell-based assay using MOP receptor-dependent suppression of adenylyl cyclase activity to determine intrinsic efficacy compared with DAMGO. The effect of ITI-333 on β-arrestin pathway signaling at MOP receptors was also investigated using the PathHunter® β-Arrestin assay (DiscoverX, Fremont, CA) using met-enkephalin as a control.

#### Dopamine D_1_ receptors

ITI-333 functional activity at dopamine D_1_ receptors was determined at a single concentration of 10 μM. Cellular agonist effects were calculated as the percent of control response to 10 μM dopamine; antagonist effects were calculated as the percent inhibition of 300 nM dopamine response in CHO cells expressing human recombinant D_1_ receptor.

#### Adrenergic α_1A_ receptors

ITI-333 functional activity on adrenergic α_1A_ receptor-dependent calcium signaling was assessed using a whole cell-based AequoZen assay system. Adrenergic α_1A_ receptor activation was detected via measurement of light emission after addition of increasing concentrations of ITI-333 or the positive control agonist phenylephrine (≤ 100 µM) in CHO-K1 cells with stable co-expression of aequorin and adrenergic α_1A_ receptors. In antagonist mode, the reversal of agonist-induced (50 nM phenylephrine) calcium response by increasing concentrations of ITI-333 (≤ 10 µM) or a positive control (tamsulosin [≤ 1 µM]) was examined.

### In vivo functional assays

#### Blockade of 5-HT_2_ agonist-induced head twitch

Functional activity of ITI-333 at 5-HT_2A_ receptors was assessed in vivo using a head twitch paradigm in adult (8–10 weeks) male C57Bl/6 mice (Jackson Labs, Wilmington, MA). Mice received a s.c. injection of ITI-333 (n = 15; 0.01–10 mg/kg) or vehicle (n = 3; 45% Trappsol in water) 30 min prior to an intraperitoneal (i.p.) injection of the 5-HT_2_ agonist 2,5-Dimethoxy-4-iodoamphetamine (DOI; 2.5 mg/kg), after which they were immediately placed in a novel cage for behavioral observation. During the 5-min observation period, head twitches (rapid side-to-side rotational head movements) were counted manually by a blinded observer. The mean number of head twitches for each treatment group was calculated. Curve fitting was performed with the XL-Fit add-on to Microsoft Excel to estimate an ID_50_ for inhibition of DOI-induced headtwitch by ITI-333, using a 4-parameter logistic fit.

#### Morphine-induced hyperactivity

Adult (8–10 weeks) male C57Bl/6 mice (Jackson Labs, Wilmington, MA) were habituated to locomotor chambers (Ohio Instruments, Hiram, OH) without treatment, and their spontaneous horizontal locomotor activity recorded for 30 min. Groups of mice (n = 5 each) then received morphine sulfate (32 mg/kg, s.c.) followed immediately by ITI-333 (0.01–3 mg/kg, s.c.) or vehicle. After 30 min, mice were returned to the locomotor chambers and the cumulative distance traveled over 120 min was measured using video-tracking software (AccuScan, AUT Solutions, Fulshear, TX). The effect of ITI-333 was calculated as the cumulative distance traveled after administration of ITI-333 as a percentage of the distance traveled by vehicle-treated mice.

#### Spontaneous locomotor activity

Adult (8–12 weeks) male Swiss-Webster mice (Hsd:ND4) were injected (subcutaneous) with either vehicle (45% hydroxypropyl-beta-cyclodextrin) or ITI-333 (0.03, 1, or 3 mg/kg) 30 min before locomotor activity assessment. All mice (n = 8/group) were non-habituated and received an intraperitoneal saline injection immediately before placement into activity test chambers (Digiscan; 40.5 × 40.5 × 30.5 cm). Horizontal activity counts, assessed by interruptions of 16 infrared beams, were measured in 10-min bins. A one-way ANOVA and planned comparisons to the vehicle control group were conducted on horizontal activity counts during the 30-min period following placement in the test chamber. Mean horizontal activity counts were fit to a linear function of dose of the descending portion of the dose–effect curve to determine the dose producing half-maximal depressant activity (ID_50_), where maximal depression equaled 0 counts per 30 min.

#### Prevention of naloxone-precipitated withdrawal from oxycodone

Adult (8–10 weeks) male C57Bl/6 mice (Jackson Labs, Bar Harbor, ME) were administered saline or oxycodone twice daily for 8 d at increasing daily doses (9, 17.8, 23.7, and 33 mg/kg, s.c. on days 1–2, 3–4, 5–6, and 7–8, respectively). On day 9, mice were injected with ITI-333 (0.3, 1, or 3 mg/kg, s.c.; n = 32 each) or vehicle (n = 16), followed 30 min later with an injection of naloxone (3 mg/kg, s.c. in saline) to precipitate abrupt opioid withdrawal. Immediately following naloxone injections, mice were observed continuously for 30 min; somatic signs of withdrawal, including jumps, wet-dog shakes, paw tremors, backing, ptosis, and diarrhea, were recorded manually by a blind observer. All behaviors were recorded as new incidences when separated by at least 1 s or interrupted by any other normal behavior. Data were analyzed with analysis of variance (ANOVA) tests followed by Tukey tests for multiple comparisons.

#### Precipitation of withdrawal from oxycodone by ITI-333

Adult (8–10 weeks) male C57Bl/6 mice (Jackson Labs, Bar Harbor, ME) were administered oxycodone as described above. On the morning of the 9th day, mice were administered oxycodone (33 mg/kg, s.c.) followed 2 h later with an injection of ITI-333 (3, 10, or 17.8 mg/kg, s.c.; n = 8 each) or vehicle (n = 8). A separate group of mice was chronically administered saline instead of oxycodone and was challenged with ITI-333 (17.8 mg/kg, s.c.; n = 8) or vehicle (n = 8) on day 9 to evaluate effects of ITI-333 alone. Thirty minutes following vehicle or ITI-333 injections on day 9, mice were individually placed in Plexiglas cages and observed for somatic signs of withdrawal as described above. Data were analyzed with ANOVA followed by Dunnett’s tests for multiple comparisons.

#### Cue-induced reinstatement of heroin self-administration

Adult (280–300 g upon arrival) male Long-Evans hooded rats (Envigo, Indianapolis, IN) were trained to self-administer heroin. Training sessions were conducted for 5 d/week for 2 h/d using test chambers equipped with two retractable levers, white cue lights positioned above each lever, a 5W house light, and a tone generator. Rats were outfitted with an indwelling catheter in the right external jugular vein using a protocol described previously (Shelton et al. 2013). Rats were trained using a fixed ratio 1 (ie, 1 infusion per lever press; FR1) reinforcement schedule on the active (right-side) lever to deliver a 0.01 mg/kg heroin infusion (0.14 ml/6 s) through the indwelling catheter. For the duration of the infusion, the tone sounded and the stimulus lights above both levers flashed at 3 Hz. Active lever presses during the infusion and inactive lever presses were without consequences. Active and inactive lever presses and activation of lights, pumps, and tones were recorded. Self-administration training continued until three criteria were met: 1) at least 12 self-administration sessions occurred; 2) at least 15 heroin infusions occurred during each of the last four sessions; and 3) at least 125 lifetime heroin infusions had been obtained.

After successful self-administration training, 12 extinction sessions (2 h/d) were conducted in which the house light was illuminated and the levers were extended but infusions were not administered and other scheduled stimuli (tone or light) did not occur. Conditions during subsequent reinstatement testing were identical to those during self-administration training, except that ITI-333 (1, 3, or 10 mg/kg, n = 12 each) or vehicle (n = 12) was administered 30 min prior to testing and heroin self-administration did not occur. Additionally, cues previously associated with heroin infusion were presented non-contingently for 6 s at the start of the reinstatement testing session (i.e., the tone sounded, the stimulus lights above both levers flashed at 3 Hz for 6 s, and the house light was off).

Data were analyzed using individual ANOVAs. If results were found significant, comparisons between groups were conducted using Tukey’s Multiple Comparison tests. Further, a paired, one-tailed t-test was conducted comparing active-lever presses during the last extinction session with those during the reinstatement test session of the vehicle group to determine if the heroin cue conditions used were capable of reinstating responding. In addition, numbers of inactive level presses (i.e., on the left side lever) occurring during the last test session were compared between groups using ANOVA. All statistical tests were conducted using Prism 7 for Macintosh (GraphPad Software, Inc., San Diego, CA), and all comparisons were considered statistically significant if *p* < 0.05.

#### Reinforcement of self-administration in heroin-maintained rats

Adult (200–300 g at the start of the study) male Sprague–Dawley rats (Charles River, Margate, Kent, UK) were trained under mild food restriction to lever press for food rewards on a FR3 schedule of reinforcement. When competent in this task, pharmacologically active doses of ITI-333 were defined in these animals by observing the effects of intravenous (i.v.) bolus injections (0.001, 0.003 and 0.01 mg/kg, i.v.) on general behavior and FR3 operant responding for food rewards (n = 3–10/group). Based on this experiment, 0.0003, 0.001, 0.003 and 0.01 mg/kg/inj (i.v.) doses were selected for the self-administration experiment (n = 8/group). Rats were surgically implanted with an indwelling jugular catheter for the i.v. self-administration experiments and trained to self-administer a low dose of heroin (0.015 mg/kg/inj in 0.9% saline) on a FR5 reinforcement schedule. Saline (0.5 ml/kg/inj, i.v.; n = 21) was the non-reinforcing control substance. The reinforcing effects of ITI-333 were investigated on a FR5 schedule of reinforcement.

The mean number of injections per session during the last 3 sessions for rats responding under an FR5 schedule of i.v. self-administration were calculated and then back-transformed and adjusted for between-animal differences. SEMs were calculated from the residuals of the statistical model. ITI-333 was compared to the first saline extinction session by Williams’ test and to heroin acquisition session by Dunnett’s test. The heroin acquisition session was compared to the first saline extinction session using multiple t-tests.

#### Reinforcement of self-administration in heroin-maintained rhesus monkeys

The potential reinforcing effects of ITI-333 were studied in 3 male and 2 female adult rhesus monkeys (*Macaca mulatta*), ranging in age from 7–20 years, using an intravenous self-administration procedure. The monkeys were housed in a room maintained on a 14/10-h light/dark cycle at 21 ± 1 °C and relative humidity of 50 ± 10%. They received primate chow (High Protein Monkey Diet; Harlan Teklad, Madison, Wisconsin), peanuts, and fresh fruit daily in the home cage in amounts adequate to maintain age-appropriate body weights.

##### Surgery

Monkeys were implanted with a chronic s.c. access port (MIDA-PU-C50; Instech Laboratories, Plymouth Meeting, PA) and an indwelling i.v. catheter (e.g., jugular or femoral vein) under anesthesia with ketamine (10 mg/kg, intramuscular) followed by isoflurane (1.5–3%, inhalation) maintenance. Monkeys were allowed at least 2 d for recovery post-surgery, during which time they were examined daily and received an injectable antibiotic (penicillin B&G, 40,000 IU/kg) and an analgesic (meloxicam, 0.2 mg/kg the first day; 0.1 mg/kg/d for up to 4 additional days).

##### Apparatus

For all studies, monkeys were seated in chairs (Macaque Restrainer model 1R-1R10; Primate Products, Inc., Immokalee, FL) that provided restraint at the neck and arms. During experimental sessions, chairs were located in ventilated, sound-attenuating chambers. Mounted on one wall of each chamber, within easy access to the seated monkeys, was a custom-made operant panel containing two response levers and associated stimulus lights. Only one of the levers (left or right) was activated for an individual monkey; the particular lever that was activated was chosen based on the behavioral history of the monkeys used in this study. Pellet dispensers were located on the outside of each chamber and, for some monkeys, completion of the response requirement resulted in the delivery of 300 mg raspberry-flavored sucrose pellets (Bio-Serv, Flemington, NJ) to a trough mounted under the lever panel. Infusion pumps (model PHM-100; MED Associates, Inc., Fairfax, VT) were also located outside the chamber and were connected to the implanted i.v. catheter with sterile tubing and a Huber point needle. The response panel and infusion pump were connected to and controlled by an interface and computerized system (MED Associates, Inc.). To maintain patency, catheters and ports were flushed and locked after each session with 2.5 ml of heparinized saline (100 U/ml; Hospira Inc., Lake Forest, IL).

##### Dose-finding study

Monkeys were previously trained to press a lever, in the presence of a distinctive visual stimulus, on a FR10 schedule to receive a food pellet. Daily sessions comprised eight 15-min cycles (2 h). A cycle began with a 10-min timeout, during which the chamber was dark and lever presses had no consequence. After the 10-min timeout was a response period, signaled by the illumination of a green light, during which monkeys could respond under the FR10 schedule for food. The response period ended and the chamber darkened after the delivery of 10 food pellets or 5 min, whichever occurred first.

ITI-333 (0.0001–0.32 mg/kg) was evaluated in two monkeys for its ability to decrease the rate of lever pressing for food. ITI-333 was administered through the implanted i.v. catheter. Tests were conducted no more often than once every four days and only so long as responding was stable, as demonstrated by the last three sessions before the test session in which drug was not administered. Response rate was averaged across cycles to obtain a mean rate for each session and then averaged across the three sessions; responding was considered stable when the rate for each individual session was > 75% of the mean rate for the three sessions.

##### Heroin (baseline) self-administration and vehicle substitution (extinction)

Monkeys (n = 4) were trained to respond under an FR 30 schedule for heroin (0.0032 mg/kg/infusion, i.v.) as described previously (Maguire et al. 2019). Thereafter, vehicle replaced heroin (i.e., extinction) for a minimum of 4 sessions and until monkeys received fewer than 8 infusions in each of 3 consecutive sessions in which the average response rate was less than 20% of the average response rate for sessions when heroin was available (for each individual monkey). In separate sessions, ITI-333 (0.01, 0.032, and 0.1 mg/kg/inf, i.v.) was substituted for vehicle. Each dose was studied for a minimum of 5 and a maximum of 10 sessions. A “priming” (noncontingent) infusion of ITI-333 was administered immediately before each session. Following assessment of each dose of ITI-333, vehicle was available for self-administration (i.e., washout) for a minimum of 4 sessions.

##### Heroin self-administration retest

After completion of sessions with the three doses of ITI-333, monkeys were tested again with heroin (0.0032 mg/kg/inf, i.v.) for a minimum of 5 sessions according to the criteria described above.

#### Potential of ITI-333 to Induce Pharmacological Tolerance/Physical Dependence on Withdrawal

Adult (200–250 g at the beginning of the study) male Sprague–Dawley rats (Charles River, Margate, Kent, UK) were tested for signs of physical dependence upon withdrawal of ITI-333, morphine, or vehicle (10% Trappsol + 1% Tween 80 in water). During the baseline phase (Day -6 to Day 0), all animals received vehicle by s.c. and p.o. routes at 08:00 h to familiarize them with dosing and handling. On day 0, rats were randomized to treatment with vehicle, ITI-333, or morphine (positive control). During the drug dosing phase (Day 1 to Day 28), ITI-333 was administered once daily and morphine was administered twice daily, as follows:

**Table:** 

Group	Treatment 1 (08:00 h)	Treatment 2 (15:00 h)
Vehicle/Vehicle	Vehicle (2 ml/kg s.c., 5 ml/kg p.o.)	Vehicle (5 ml/kg p.o.)
ITI-333/Vehicle	ITI-333 (0.3 mg/kg s.c.); Vehicle (5 ml/kg p.o.)	Vehicle (2 ml/kg s.c.)
ITI-333/Vehicle	ITI-333 (3 mg/kg s.c.); Vehicle (5 ml/kg p.o.)	Vehicle (2 ml/kg s.c.)
Vehicle/Morphine	Vehicle (5 ml/kg s.c.); Morphine (30 mg/kg p.o.)	Morphine (30 mg/kg p.o.)

Rats received their final treatment doses on Day 28; during the subsequent 7-day drug withdrawal phase (Day 29 to Day 35), no treatments were administered. Throughout each phase of the study, all animals were assessed once daily for behavioral, physical, and physiological signs including body weight, food intake, water intake, and temperature. Statistical tests for comparisons against the vehicle/vehicle group were performed using Williams’ test (ITI-333-treated groups) or multiple t-tests (morphine-treated group).

### Pulmonary assessment

Adult (9–10 weeks, 270–332 g at the start of the study) male Sprague–Dawley rats (n = 6/group; Charles River) were habituated for 2 d in a plethysmograph chamber prior to the experiment. On the testing day, each rat was placed in the chamber and baseline respiratory parameters were obtained for 5 min. Rats were then removed from the chamber and administered ITI-333 (0.3, 1, 3 mg/kg, s.c.) or vehicle. Rats were returned to the plethysmograph chamber immediately following dosing, and respiratory parameters—including respiratory rate, tidal volume, and minute volume—were measured during 5-min intervals at 30 min (± 3 min), 1, 2, and 4 h (± 5 min). Values for the ITI-333-treated group were compared to vehicle controls and baseline using a two-way repeated measures ANOVA followed by a Bonferroni Test for Multiple Comparisons (SigmaStat v.2.03). P values < 0.05 were considered statistically significant.

### Measurement of GI propulsion

GI peristalsis was measured using a motility indicator (transit of a 10% suspension of activated charcoal in 0.25% methylcellulose) administered orally 15 min posttreatment with vehicle, ITI-333, or morphine (positive control). Adult (8 weeks of age, 218–246 g) male Sprague Dawley rats (Charles River) were given a single s.c. dose of vehicle, ITI-333 (0.3, 1, or 3 mg/kg in vehicle), or morphine (5 mg/kg in sterile water; n = 8/group). Rats were euthanized 30 min after charcoal administration and intestines were removed; the length of the intestine (pyloric sphincter to caecum) and the distance traveled by the charcoal as a fraction of that length were measured for each rat. In a second experiment, the ability of ITI-333 to block morphine-induced inhibition of GI motility in rats was studied by administering a single dose of ITI-333 (3 mg/kg, s.c.) either 30 min after or 60 min prior to administration of morphine (5 mg/kg). Values for charcoal motility were expressed as a percent effect (distance traveled divided by the total length of the intestines). Mean values were calculated for each group and percent effect was calculated using the following formula:

$$\%effect=\frac{\left(mean\ value\ for\ controls\right)-\left(mean\ value\ for\ treated\right)}{mean\ value\ for\ controls} \times 100$$

Group comparisons were performed using ANOVA with Tukey HSD test for multiple comparisons. P values < 0.05 were considered statistically significant.

## Results

### Receptor binding assays

ITI-333 displayed appreciable binding affinity—as defined by ≥ 40% binding at the test concentration—only at 5-HT_2A_, MOP, D_1_, D_2_, and α_1A_ receptors (Table 1). No significant binding was detected at any other targets studied, including other opioid receptors (κ, δ, or nociception opioid receptors [NOP]) (SupplementalFig. 1).

**Table 1:** Receptor binding selectivity of ITI-333

Binding Assay	% Inhibition of Control Specific Binding^a^
5-HT_2A_*(h)*	90
μ (MOP)*(h)*	86
α_1A_*(h)*	64
D_2S_*(h)*	50
D_1_*(h)*	40

In vitro binding selectivity was measured against a broad specificity profile panel. Shown here are receptor interactions with > 40% binding at a 100 nM concentration of ITI-333 (of a total of 44 substrates evaluated)

^a^Values are expressed as the percent inhibition of specific binding and represent the average of replicate tubes at the specified drug concentration

### In vitro concentration–response assays

The binding affinity of ITI-333 to 5-HT_2A_, MOP, D_1_, D_2_, and α_1A_ receptors was characterized in more detail with cell-based concentration–response assays as shown in Table 2. ITI-333 displayed high-affinity binding to 5-HT_2A_ (K_i_ = 8.3 nM), adrenergic α_1A_ (K_i_ = 28 nM), dopamine D_1_ (K_i_ = 50 nM), and MOP (K_i_ = 11.0 nM) receptors. ITI-333 displayed modest binding to D_2_ (K_i_ = 160 nM) receptors.

**Table 2:** ITI-333 Binding to serotonin, dopamine, opioid, and adrenergic receptors

Assay	Dose Range	IC_50_ (nM)	K_i_ (nM)
5-HT_2A_*(h)* (agonist radioligand)	20 pM–50 nM	11	8.3
MOP*(h)* (agonist radioligand)	1 nM–2.19 μM	28	11
α_1A_*(h)* (antagonist radioligand)	0.3 nM–10 μM	56	28
D_1_*(h)* (antagonist radioligand)	0.46 nM–1 μM	120	50
D_2S_*(h)* (antagonist radioligand)	1.37 nM–3 μM	490	160

### Cell-based assays for functional activity of ITI-333

The functional activity of ITI-333 at each of major binding targets identified in Table 2 was further investigated. At human recombinant 5-HT_2A_ receptors, ITI-333 acted as an antagonist, suppressing calcium (Ca^2+^) flux elicited by the serotonin agonist α-methylserotonin with an apparent dissociation constant (K_B_) of 9.5 nM. ITI-333 had no agonist activity (i.e., did not stimulate Ca^2+^ flux) at 5-HT_2A_ receptors when tested in the same cell line. At the dopamine D_1_ receptor, ITI-333 blocked forskolin-induced cAMP accumulation (i.e., was an antagonist) in cells expressing human D_1_ receptors (K_B_ = 1.4 µM) while displaying no agonist activity when tested alone. Finally, ITI-333 demonstrated antagonist activity at adrenergic α_1A_ receptors by inhibiting phospholipase C (PLC)-associated Ca^2+^ signaling with an IC_50_ of 33.4 nM; the K_B_ value for ITI-333 was calculated as 7.74 nM. ITI-333 demonstrated no agonist α_1A_ activity at concentrations up to 10 μM.

Ligands acting at the MOP receptor can signal through either G-protein-dependent signaling pathways (Gi/o) to inhibit accumulation of cAMP in cells or via β-arrestin-dependent pathways (Bohn et al. 2003). ITI-333 acted as a partial agonist at human recombinant MOP (hOP3) receptors expressed in CHO-K1 cells (Table 3), inhibiting cAMP accumulation elicited by forskolin (10 µM) with a maximal effect, or intrinsic efficacy, equal to 22% of that of the full MOP agonist DAMGO and an EC_50_ of 64.45 nM. In comparison, buprenorphine, a well-characterized partial agonist at MOP receptors, showed higher intrinsic activity than ITI-333 under the same assay conditions, with an intrinsic efficacy of 78% of DAMGO and an EC_50_ of 0.95 nM. In antagonist assays employing the same cell system, ITI-333 reversed the inhibition of cAMP accumulation elicited by 10 nM DAMGO with an IC_50_ of 641.5 nM, as compared to naloxone with an IC_50_ of 5.8 nM. The K_B_ for ITI-333 was calculated as 71.4 nM. Thus, ITI-333 acted as a partial agonist at MOP receptors with lower intrinsic activity than buprenorphine at hOP3 receptors and less potency than naloxone as an antagonist in the hOP3 cell system.

**Table 3:** Agonist and antagonist properties of ITI-333 measured in human MOP receptors expressed in CHO-K1 cells in vitro

Compound	Assay Format	EC_50_ (nM)	IC_50_ (nM)	Apparent Dissociation Constants K_B_ (nM)	Max Response (%)
DAMGO	Agonist	1.56			102.4
Naloxone	Antagonist		5.8	0.65	88.27
Buprenorphine	Agonist	0.95			77.71
ITI-333	Agonist	64.45			21.56
ITI-333	Antagonist		641.5	71.44	71.66

Max response defined as the greatest agonist activity (using 1 µM DAMGO as a control) or antagonist activity (using reversal of DAMGO-inhibited cAMP production)

MOP-induced analgesia has been associated with Gi/o-dependent signaling, whereas certain adverse effects including respiratory depression and adverse GI effects are thought to be mediated through activation of the β-arrestin signaling pathway (Bohn et al. 2000; Raehal et al. 2005). ITI-333 was found to have no significant activity as a β-arrestin agonist (EC_50_ > 10 µM; Supplemental Table 1). Rather, ITI-333 antagonized MOP receptor-dependent β-arrestin signaling (IC_50_ = 0.19 µM). In contrast, the full opioid agonist met-enkephalin acted as an agonist of β-arrestin-dependent signaling pathways, stimulating β-arrestin signaling with an EC_50_ of 0.08 µM.

### In vivo functional assays

#### ITI-333 inhibits 5-HT_2A_ receptor-dependent activity in mice

In a mouse head twitch paradigm, ITI-333 dose-dependently reduced the number of head twitches elicited by the 5-HT_2A_ receptor agonist DOI with an ID_50_ of 0.22 ± 0.3 mg/kg (SupplementalFig. 2). These data provide in vivo support for the in vitro cell-based receptor assays indicating that ITI-333 acts as a functional antagonist at 5-HT_2A_ receptors.

#### ITI-333 dose-dependently reduces morphine-induced hyperactivity in mice

**Fig. 2:**
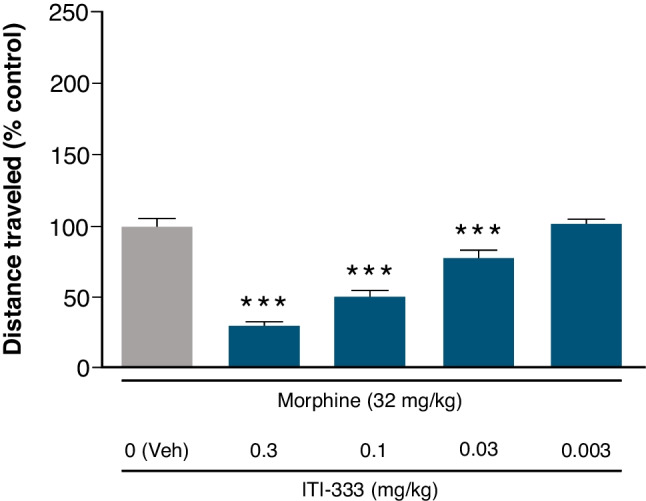
ITI-333 dose-dependently reduces morphine-induced hyperactivity in mice. Distance traveled (cm) was quantified for each group (n = 5 mice/group) and is expressed as the mean (± SEM) percentage of the control (Morphine/Veh) group. ***p < 0.001 vs Morphine/Veh. Veh, vehicle

Treatment of mice with a high dose of morphine (32 mg/kg, s.c.) results in increased locomotor activity (hyperactivity) that can be suppressed by opioid receptor antagonists (e.g., naloxone). At doses as low as 0.03 mg/kg significantly reduced morphine-induced hyperactivity (*F*_(3,56)_ = 55.22; *p* < 0.001 vs morphine alone; Fig. 2). The greatest attenuation of morphine-induced hyperactivity was seen in the highest ITI-333 dose group (0.3 mg/kg); at this dose, ITI-333 was found to have no sedative effects compared to vehicle on spontaneous locomotor activity in a separate study (SupplementalFig. 3). The ability of ITI-333 to block behavioral effects of a high dose of morphine in vivo is consistent with the properties of ITI-333 as a partial MOP receptor agonist established in cell-based receptor assays.

#### ITI-333 dose-dependently reduces naloxone-precipitated withdrawal in oxycodone-treated mice

**Fig. 3:**
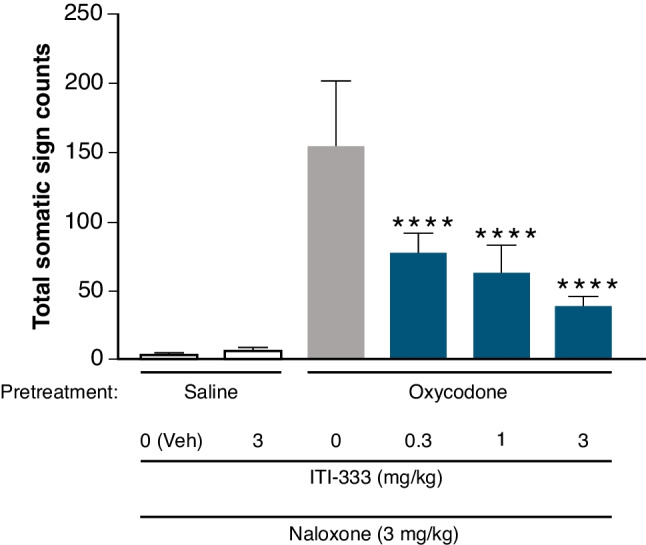
ITI-333 dose-dependently reduces naloxone-precipitated withdrawal in oxycodone-treated mice. Mice were observed for somatic signs of withdrawal including jumps, wet dog shakes, paw tremors, backing, ptosis, and diarrhea for 30 min. Data are expressed as means (± SEM) for 8 mice/ group. ****p < 0.0001 vs the group pretreated with oxycodone and tested with veh/naloxone (Tukey’s post hoc multiple comparisons test). Veh, vehicle

In mice made physically dependent on the opioid analgesic oxycodone, naloxone precipitated somatic signs of withdrawal (including jumps, wet-dog shakes, paw tremors, backing, ptosis, and diarrhea) measured over 30 min. At all doses tested, ITI-333 significantly decreased the total number of somatic signs precipitated by naloxone (*F*_(5,42)_ = 50.32, *p* < 0.0001 [ANOVA]). Post-hoc analysis indicated significant decreases in the total number of somatic signs compared with mice treated with oxycodone alone at each dose of ITI-333 (*p* < 0.05 [Tukey’s multiple comparison test]; Fig. 3).

#### Precipitation of withdrawal from oxycodone by ITI-333 in mice

**Fig. 4:**
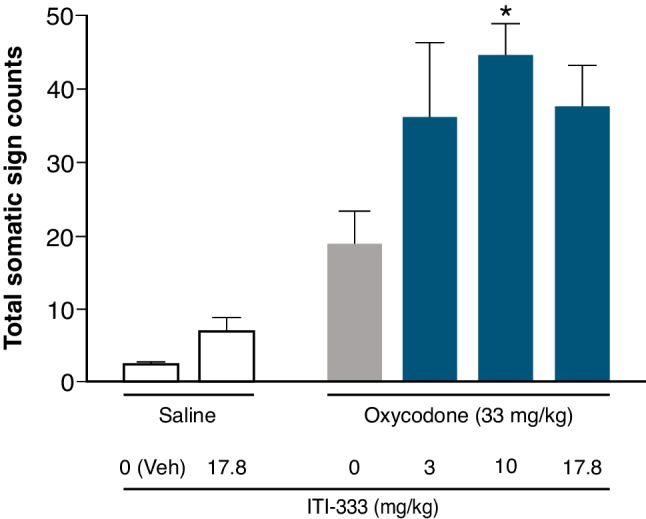
Precipitation of withdrawal from oxycodone by ITI-333 in mice**.** Mice chronically treated with oxycodone or saline were challenged with ITI-333 or vehicle (Veh) and observed for manifestation of somatic signs of withdrawal (including jumps, wet dog shakes, paw tremors, backing, ptosis, and diarrhea). Bars represent the mean (± SEM) for 8 mice/treatment group. *p < 0.05 vs the group chronically administered oxycodone and challenged with vehicle

There was a significant effect of treatment on total number of somatic signs of withdrawal (F_(5, 42)_ = 4.39, *p* < 0.001 [one-way ANOVA]). Post hoc analyses revealed that mice chronically treated with oxycodone and challenged with 10 mg/kg ITI-333 on test day had a greater number of total withdrawal signs relative to mice chronically treated with oxycodone and challenged with vehicle on test day (*p* < 0.05 [Dunnett’s test for multiple comparisons] Fig. 4). The effect of 10 mg/kg ITI-333 on total somatic withdrawal signs was primarily driven by an increase in wet dog shakes (*p* < 0.05 [Dunnett’s test for multiple comparisons] Supplementary Table 2). Importantly, in mice chronically treated with saline, ITI-333 did not have a significant effect on somatic signs of withdrawal relative to vehicle.

#### ITI-333 reduces cue-induced reinstatement of heroin self-administration in rats

**Fig. 5:**
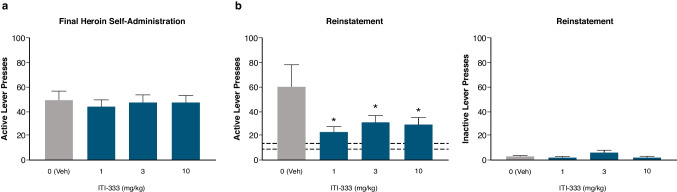
ITI-333 reduces cue-induced reinstatement of heroin self-administration in rats**. a**) Number of active lever presses during the last day of self-administration were not significantly different across groups **b**) active (left) and inactive (right) lever presses were measured during heroin cue-induced reinstatement. Dashed horizontal lines indicate the range of the means of active lever presses across dosage groups occurring during the last extinction session. Data are presented as mean (± SEM) lever presses for 12 rats/group. *p < 0.05 vs vehicle (Tukey’s multiple comparison test). Veh, vehicle

After heroin-reinforced lever pressing was established, and then extinguished, cue-induced reinstatement was conducted. During the final session of self-administration, active lever presses were similar across all groups (F_(3,43)_ = 0.108; *p* = 0.9552; Fig. 5a). The number of active lever presses during the last session of extinction in the vehicle-treated group (9.83 ± 3.16) increased significantly to 60.33 ± 18.07 (*t* = 2.824, df = 11; *p* < 0.01) during the reinstatement test, confirming cue-reinstated responding in this group (Fig. 5b). All doses of ITI-333 significantly decreased cue-reinstated responding relative to the vehicle group (*t* = 2.582, df = 43; *t* = 2.042, df = 43; *p* = 0.0473 for 3 mg/kg dose and *t* = 2.159). Inactive lever presses during the reinstatement test were low overall and not significantly different between treatment groups (*F*_(3,43)_ = 2.53; *p* = 0.0697).

#### ITI-333 does not reinforce lever pressing in heroin-maintained rats

**Fig. 6:**
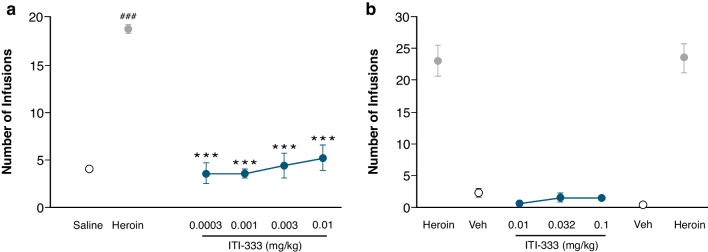
ITI-333 does not reinforce lever pressing in heroin-maintained rats or monkeys**. a**) Rats (n = 21 rats total, 8 rats per ITI-333 dose group) and **b**) monkeys (n = 4) were trained to self-administer heroin and then maintained on a low dose of heroin. Saline was the non-reinforcing control substance. Reinforcing effects of ITI-333 were tested at a range of doses, up to the limit of solubility, in a self-administration experiment. ###*p* < 0.001 heroin acquisition (Heroin) versus saline extinction (Saline); ***p < 0.001 ITI-333 versus Heroin. Statistical analyses were not performed on data from the monkey self-administration experiment. Data are presented as mean (± SEM). Veh, vehicle

Across a 30-fold range of doses, ITI-333 did not support lever pressing on a FR5 schedule of reinforcement (Fig. 6a). Rats were observed to self-administer solutions of 0.0003, 0.001, 0.003, and 0.01 mg/kg/inj ITI-333 at rates of 3.17 ± 1.13, 3.29 ± 0.46, 3.99 ± 1.18, and 4.87 ± 1.27 inj/session, respectively, compared with 4.08 ± 0.24 inj/session for saline. The number of inj/session for all groups that self-administered ITI-333 was significantly lower than the number of inj/session received by the same rats when self-administering heroin during the final day of maintenance (0.15 mg/kg/inj; 19.38 ± 0.32 inj/session; *p* < 0.001 all). The responding of the animals during heroin reinstatement was significantly lower than responding during heroin acquisition (*p* < 0.001), indicating that ITI-333 may have produced some aversion to lever pressing.

#### ITI-333 does not reinforce lever pressing in heroin-maintained rhesus monkeys

The potential reinforcing effects of ITI-333 were further investigated in adult rhesus monkeys. In a dose range-finding study, doses of ITI-333 up to 0.32 mg/kg/inf i.v. were examined and did not affect responding for food (SupplementalTable 3). In the heroin self administration study, substitution of vehicle for heroin (0.0032 mg/kg/inf) markedly decreased the average number of infusions received per session by ten-fold, from 23.2 ± 2.3 to 2.3 ± 0.8. When ITI-333 was substituted for vehicle, the number of infusions received remained low and was not different from vehicle (Fig. 6b). The average number of infusions of ITI-333 received per session was 0.6 ± 0.2, 1.6 ± 0.6, and 1.5 ± 0.3 (0.01, 0.032, and 0.1 mg/kg/inf, respectively). Following washout sessions in which vehicle was available for self-administration (average 0.3 ± 0.2 inf/session), monkeys were again tested with heroin; the average number of infusions per session (23.6 ± 2.3) was similar to previous rates with heroin self-administration, confirming the reliability of responses to a reinforcing compound. These results demonstrate that ITI-333, tested at a dose range that did not disrupt operant responding for food, had no apparent positive reinforcing effects in a nonhuman primate species.

#### Potential of ITI-333 to induce pharmacological tolerance/physical dependence on withdrawal

##### Behavioral observations

Repeat dosing of rats with morphine (30 mg/kg, p.o., b.i.d.) was associated with behavioral effects typical for that drug (data not shown). The predominant behavioral effects induced by morphine during the on-dose phase (28 days) were Straub tail, jumping, digging, increased body tone, increased locomotor activity, explosive movements, and exophthalmos. Abrupt cessation of morphine administration on Day 28 was also associated with clinical signs indicating the presence of dependence; behavioral effects noted were piloerection, ataxia/rolling gait, wet dog shakes, and pinched abdomen. By Day 35, rearing was the only behavior or physical sign reported with high incidence in the rats that had previously received morphine (data not shown). Thus, repeated administration of morphine was associated with expected behavioral changes indicative of tolerance; upon withdrawal of morphine, a clear syndrome of physical dependence was observed.

The predominant behavioral and physical effects induced by repeated dosing of ITI-333 (0.3 mg/kg s.c., q.d.) were hunched posture, Straub tail, and piloerection; effects were similar for rats receiving the 3 mg/kg dose (i.e., hunched posture, subdued behavior, Straub tail, tail rattle and piloerection). A similar profile of behavioral and physical signs to that observed during the on-dose phase was also observed following abrupt cessation of ITI-333 (0.3 and 3 mg/kg s.c.) on Day 28. Rearing and increased body tone were not observed during the on-dose phase for ITI-333 (0.3 mg/kg), but were significantly increased during the withdrawal phase in rats receiving 0.3 and 3 mg/kg ITI-333. Thus, repeated administration of ITI-333 at either dose did not produce tolerance during 28 days of dosing. Furthermore, upon withdrawal, a similar but decreasing profile of behavioral and physical signs relative to the on-dose phase was observed at the highest dose.

##### Physiological effects

**Fig. 7:**
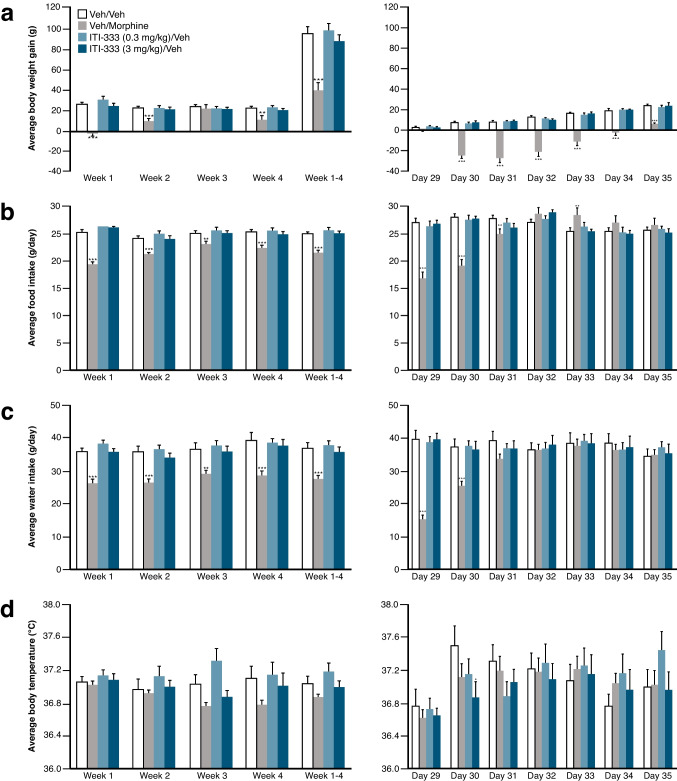
Body weight, food intake, water intake and body temperature of rats receiving repeated doses of morphine or ITI-333 and after abrupt drug withdrawal**. a**) Body weight, **b**) food intake, **c**) water intake, and **d**) temperature were measured for rats receiving repeated doses of morphine (30 mg/kg, p.o., bid) or ITI-333 (0.3 or 3 mg/kg, s.c., qd) over 28 days (left panels) and during 7 days (Day 29–35) after cessation of drug administration (right panels. *p < 0.05; **p < 0.01; ***p < 0.001 vs Veh/Veh group. Data are presented as mean (± SEM) for 10 rats in each treatment group. Veh, vehicle

Repeat dosing of morphine (30 mg/kg, p.o., b.i.d.), was associated with changes in body weight, food and water intake, rectal temperature, and clinical signs consistent with the development of tolerance and withdrawal-induced dependence (Fig. 7). In contrast, repeat dosing of ITI-333 (0.3 or 3 mg/kg s.c., daily) produced no significant effect on body weight, food and water intake, or body temperature during the on-dose or withdrawal phases in rats.

#### ITI-333 does not cause respiratory depression in conscious rats

Potent MOP agonists, through their interactions with receptors expressed on respiratory neurons in the brainstem, depress ventilation. ITI-333 (0.3, 1, and 3 mg/kg, s.c.) did not produce statistically significant changes in respiratory rate, tidal volume, or minute volume in conscious male rats when compared to vehicle controls (Supplemental Fig. 4).

#### ITI-333 blocks morphine-induced reductions in GI motility and propulsion in rats

**Fig. 8:**
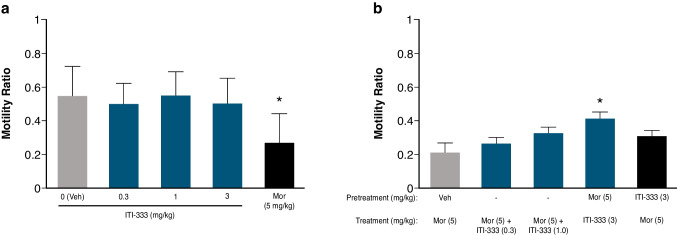
Effects of ITI-333 on GI motility in rats. GI motility was measured as the intra-intestinal distance traveled by a bolus of activated charcoal. **a**) Motility was significantly reduced by pretreatment with morphine (5 mg/kg), but was unaffected by pretreatment with ITI-333 (0.3, 1, or 3 mg/kg). *p < 0.05 vs vehicle-treated rats (grey bar). **b**) ITI-333 (3 mg/kg) administered 30 min after morphine pretreatment blocked morphine-induced inhibition of GI motility. *p < 0.05 vs rats pretreated with vehicle and treated with morphine (grey bar). Data are presented as the mean (± SEM) ratio between distance traveled by charcoal and intestine length for 8 rats/group. Mor, morphine; Veh, vehicle

Opioid agonists can decrease GI motility, causing constipation, nausea, bloating, ileus, and sometimes GI pain. Compared to the vehicle control group, morphine produced a 51% decrease in GI motility (*p* < 0.05), whereas ITI-333 alone at all doses tested produced no statistically significant or biologically relevant changes (Fig. 8a). Further, morphine-induced inhibition of GI propulsion was numerically antagonized by concurrent administration of two doses of ITI-333 (0.3 and 1 mg/kg) though differences were not statistically significant (Fig. 8b). When ITI-333 (3 mg/kg, s.c.) was administered 30 min after morphine, GI motility was significantly (*p* < 0.05) increased (+ 95%; *p* < 0.05) compared to morphine control alone. If the same dose of ITI-333 was administered 60 min after morphine, GI motility was not significantly increased (+ 43%; *p* > 0.05) compared with the morphine control group.

## Discussion

We report the pharmacological profile of ITI-333, a chemically novel, brain-penetrant, potent small molecule possessing a unique combination of high-affinity binding to 5-HT_2A_ and MOP receptors with minimal binding to other opioid receptors (i.e., κ, δ, or NOP) and lower affinity for dopamine D_1_ and adrenergic α_1A_ receptors. Pharmacologically, ITI-333 is an antagonist at 5-HT_2A_ and D_1_ receptors. At MOP receptors, it is a low intrinsic efficacy, biased partial agonist, acting as an antagonist in the presence of high levels of MOP receptor activity and as an agonist in the presence of low levels of MOP receptor activity. ITI-333 inhibited naloxone-precipitated oxycodone withdrawal symptoms in mice, indicating utility of the compound in easing the physical consequences of opioid withdrawal that, in humans, often drive relapse of opioid taking. Relapse after periods of abstinence poses a significant barrier to successful treatment of drug addiction and may be precipitated by a variety of events including stress, exposure to environmental stimuli previously associated with opioids, or limited reexposure to opioids. ITI-333 pretreatment potently inhibited cue-induced relapse of heroin self-administration in heroin-trained rats, suggesting it is capable of suppressing cue-dependent reinstatement of opioid use. The efficacy of ITI-333 in rodent models of opioid withdrawal and relapse supports further study in humans as a potential agent for treatment of OUD.

In cell-based assays, ITI-333 is a biased MOP agonist, acting as an agonist of cAMP-dependent signaling cascades. In contrast, ITI-333 lacks detectible agonism of MOP signaling via β-arrestin, which has been proposed to underlie significant liabilities of opioid drugs (Bohn et al. 2000; Raehal et al. 2005). Although the predictive value of β-arrestin activity for assuming abuse liability and other opioid-associated side effects of new classes of opioid compounds is complex and still under study (for review, see Conibear and Kelly 2019), the addictive liability of a number of current and novel opioid compounds, including their propensity to induce GI and respiratory slowing, elicit physical and psychological dependence, and to support intravenous self-administration in animals is correlated with their affinity for signaling via β-arrestin (Raehal et al. 2005; Schmid et al. 2017).

In the current study, rodents treated acutely with ITI-333 at dose levels exceeding requirements for efficacy in models of pain and opioid abuse did not show GI slowing or changes in pulmonary function. In rats chronically treated with similar doses of ITI-333, signs of physical dependence or tolerance did not develop, even after abrupt withdrawal. Further, rats and monkeys trained to self-administer heroin failed to self-administer ITI-333 at doses up to the solubility limits of intravenous solutions, suggesting a low propensity for abuse. Together, these data support the conclusion that ITI-333 possesses a low liability for abuse and peripheral side effects at dose levels efficacious for inhibiting opioid withdrawal signs and opioid relapse/reinstatement. Future studies should also characterize the abuse potential and preclinical efficacy of ITI-333 in female rodents, as the current study was conducted in male rodents. The abuse potential of ITI-333 will also need to be thoroughly studied in humans.

ITI-333 displays high-affinity binding to and functional activity in vitro and in vivo as an antagonist of 5-HT_2A_ receptors. 5-HT_2A_ receptor antagonism is unique among currently available treatments for OUD or non-opioid treatments for mitigation of physical opioid withdrawal symptoms. The role of serotonin in mood disorders is well characterized, and post-synaptic blockade of 5-HT_2A_ receptors may contribute to antidepressant effects (Celada et al. 2004). It has been postulated that hyperkatifeia, an enhanced intensity of negative emotional or motivational effects associated with withdrawal of drugs of abuse, including opioids, contributes to drug-seeking behavior and substance use disorder (Koob 2020). The 5-HT_2A_ receptor antagonist mirtazapine has demonstrated antidepressant-like and anxiolytic-like behaviors in animal models of cocaine withdrawal (Barbosa Méndez and Salazar-Juárez 2019), and it is speculated that a 5-HT_2A_ receptor antagonist might attenuate depression and anxiety associated with opioid withdrawal (Kang et al. 2008; Graves et al. 2012). Similarly, opioid dependence and withdrawal are associated with substantial sleep disturbances; even patients maintained on methadone or buprenorphine experience highly impaired sleep (Dunn et al. 2018). 5-HT_2A_ receptor antagonism has been associated with improved sleep maintenance (Vanover and Davis 2010); thus, ITI-333 may improve sleep and indirectly improve mood and other health-related outcomes associated with OUD treatment. Attenuation of the dysphoria and disturbances of sleep and mood associated with opioid withdrawal may be possible with the unique pharmacological actions of ITI-333.

In conclusion, ITI-333 possesses a unique combination of properties as a potent 5-HT_2A_ receptor antagonist and a biased, partial agonist at MOP receptors. Functionally, ITI-333 dose-dependently suppresses physical symptoms of precipitated opioid withdrawal and reinstatement of heroin responding, suggesting promising potential for utility in easing opioid withdrawal and relapse in humans. These actions of ITI-333 were observed in animals without apparent tolerance or physical dependence after chronic dosing and without alterations in GI or pulmonary function/parameters. ITI-333 was not self-administered by heroin-maintained rats or rhesus monkeys. Currently, ITI-333 is poised to enter human clinical evaluation in Phase I safety trials with the purpose of developing this compound as a treatment for OUD, associated comorbid dysphoric symptoms, and pain.

### Supplementary Information

Below is the link to the electronic supplementary material.

Supplementary file1 (DOCX 270 KB)
