# Supplementary material for: Pharmacologic profile of ITI-333: a novel molecule for treatment of substance use disorders
Source: Psychopharmacology (Berl). 2024 May 6;241(7):1477–90. doi: 10.1007/s00213-024-06578-w (PMC11199232; doi:10.1007/s00213-024-06578-w)
Supplement: Supplementary file 1 — Supplementary file1 (DOCX 270 KB) [file 213_2024_6578_MOESM1_ESM.docx]

**SUPPLEMENTARY INFORMATION****: Pharmacologic Profile of ITI-333: A novel molecule for treatment of substance use disorders**

Gretchen L. Snyder ^1*^, Peng Li^1^, Terry Martin^1^, Lei Zhang^1^, Wei Yao^1^, Hailin Zheng^1^_,_ David R. Maguire^2,3^, Lisa R Gerak^2,3^, Kimberly E. Vanover ^4^, Charles P France^2,3^, and Robert Davis^1^

Affiliations:

^1^Intra-Cellular Therapies Inc., 430 East 29^th^ Street, Suite 900, New York, NY 10016;

^2^ University of Texas Health Science Center at San Antonio; 7703 Floyd Curl Drive (Mail Code 7764) San Antonio, TX 78229-3900;

^3^Addiction Research, Treatment and Training Center of Excellence, University of Texas Health Science Center at San Antonio;

^4^Current Address: Engrail Therapeutics, San Diego, CA 92130

*Corresponding Author:

Gretchen L. Snyder Ph.D.

Vice President, Biology

Intra-Cellular Therapies Inc

430 East 29^th^ Street, Suite 900

New York, NY 10016

Phone: 646-440-9377

E-mail: gsnyder@itci-inc.com

Journal: *Psychopharmacology*

**Supplemental Table 1 Profile of ITI-333’s impact on MOP receptor-dependent β-arrestin signaling using PathHunter® GPCR Arrestin Biosensor Assays**

| **Compound** | **Assay Format** | **EC_50_ (µM)** | **IC_50_ (µM)** | **Hill Coefficient** | **Max Response (%)** |
| --- | --- | --- | --- | --- | --- |
| Met-enkephalin | Agonist | 0.08 |  | 1.24 | 101.97 |
| Naloxone hydrochloride | Antagonist |  | 0.01 | 1.16 | 100.97 |
| ITI-333 | Agonist | >10 |  |  | 0 |
| ITI-333 | Antagonist |  | 0.19 | 1.00 | 101.85 |

Data were normalized to the maximal and minimal response observed in the presence of control compound and vehicle, respectively. For antagonist assays, data were normalized to the maximal and minimal response observed in the presence of 0.2 μM met-enkephalin and vehicle.

**Supplemental Table 2 Effects of ITI-333 on individual withdrawal signs in mice.** Mice chronically treated with oxycodone or saline were challenged with ITI-333 or vehicle (Veh) and observed for manifestation of somatic signs of withdrawal. Each point represents mean (±SEM) for 8 mice/treatment group. *p<0.05 vs the group chronically administered oxycodone and challenged with vehicle. Oxy, oxycodone; Veh, vehicle.

| **Treatment** | | **Somatic Signs of Withdrawal** | | | | |
| --- | --- | --- | --- | --- | --- | --- |
| **Chronic** | **Challenge** | **Jumps** | **Head Shakes** | **Paw Tremors** | **Wet Dog Shakes** | **Backing** |
| Saline | Veh | 0 ± 0 | 0.4 ± 0.2 | 0.6 ± 0.3 | 1.0 ± 0.4 | 0 ± 0 |
| Saline | ITI-333 (17.8 mg/kg) | 0.1 ± 0.1 | 0.1 ± 0.1 | 0.4 ± 0.3 | 4.8 ± 1.6 | 0 ± 0 |
| Oxy | Veh | 0 ± 0 | 1.8 ± 0.8 | 4.5 ± 0.8 | 11.1 ± 3.2 | 1.5 ± 1.4 |
| Oxy | ITI-333 (3 mg/kg) | 0 ± 0 | 0.1 ± 0.1 | 2.3 ± 0.9 | 33.4 ± 10.4 | 0.5 ± 0.4 |
| Oxy | ITI-333 (10 mg/kg) | 0 ± 0 | 0.3 ± 0.2 | 3.0 ± 1.0 | 40.6 ± 4.5* | 0.6 ± 0.3 |
| Oxy | ITI-333 (17.8 mg/kg) | 4.1 ± 2.7 | 0.4 ± 0.2 | 4.1 ± 1.2 | 28.3 ± 4.1 | 0.6 ± 0.3 |

**Supplemental Table** **3 Effects of ITI-333 (i.v.) on responding for food by rhesus monkeys**

| **ITI-333 Dose (mg/kg)** | **Response Rate (responses per second)^a^** |
| --- | --- |
| 0 (Vehicle) | 0.95 ± 0.03 |
| 0.0001 | 0.94 ± 0.01 |
| 0.00032 | 0.98 ± 0.08 |
| 0.001 | 0.86 ± 0.11 |
| 0.0032 | 0.92 ± 0.11 |
| 0.01 | 0.97 ± 0.21 |
| 0.032 | 0.99 ± 0.22 |
| 0.1 | 0.98 ± 0.24 |
| 0.32 | 0.95 ± 0.12 |

^a^The mean (±1 SEM) rate of responding averaged across 8-cycle sessions and then averaged across two monkeys responding under an FR10 schedule for food

**Supplemental Fig. 1 Broad selectivity screen of 44 neurotransmitter receptors, enzymes, and channels tested for binding to ITI-333 (0.1 μM)**

**
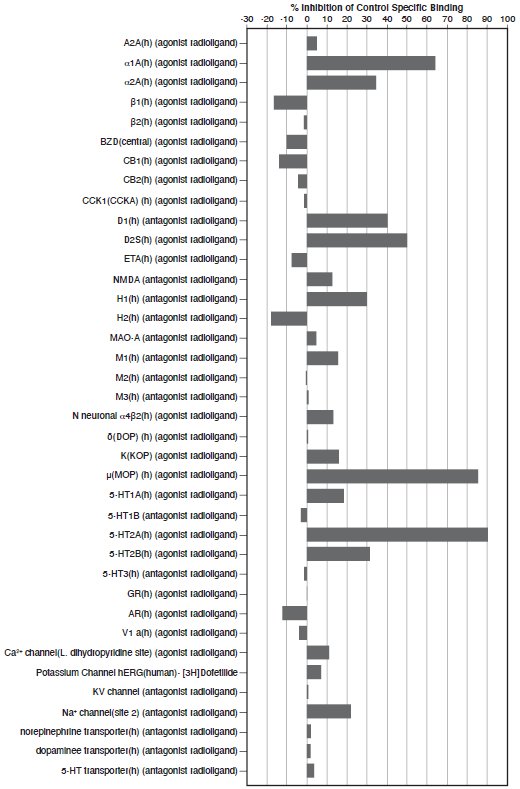
**

Results demonstrating inhibition or stimulation >50% were considered significant

**Supplemental Fig. 2 ITI-333 inhibits 5-HT2A receptor-dependent activity in mice.** The number of head twitches during a 5-min period was counted following an intraperitoneal injection of the 5-HT2 agonist 2,5-Dimethoxy-4-iodoamphetamine (DOI). Data are expressed as mean and SEM for each group (n=1-3 mice/group). Curve fitting was performed to estimate ID_50_ for inhibition of DOI-induced head twitch by ITI-333. Veh, vehicle.


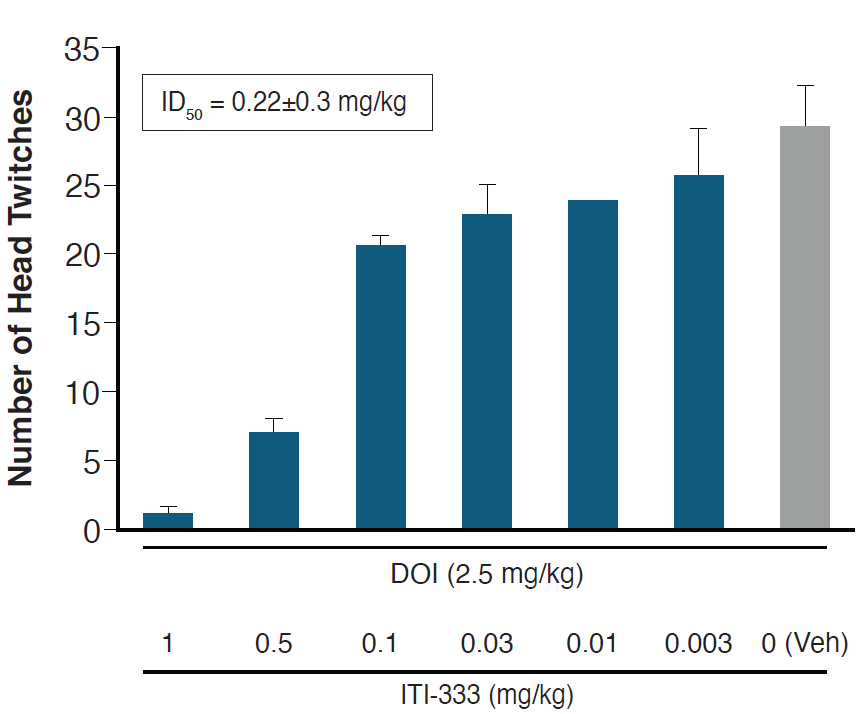


**Supplemental Fig. 3 ITI-333 (0.3 mg/kg) does not impact spontaneous locomotor activity in mice.** Horizontal activity counts (reported as the average activity during a 10-min bin) were measured across a 30-min period following subcutaneous injection of ITI-333 or vehicle. Data are expressed as mean and SEM for each group (n=8 mice/group). Data were fit to a linear function to determine the ID50 for suppression of activity by ITI-333. * p<0.05 vs vehicle (planned contrasts). Veh, vehicle.

**
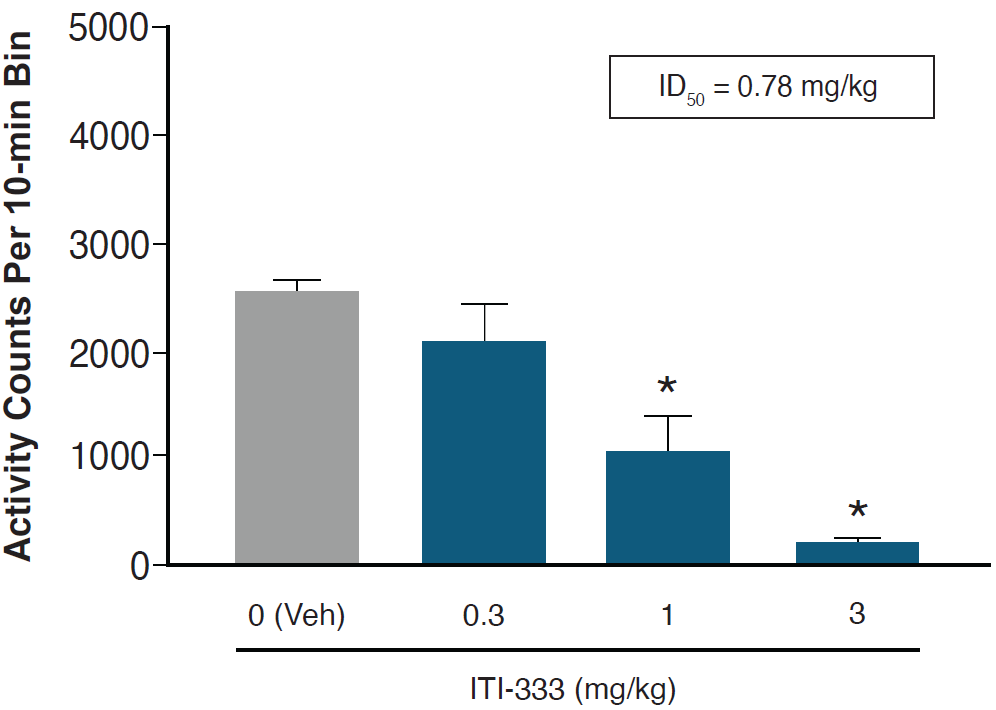
**

**Supplemental Fig. 4 ITI-333 does not cause respiratory depression in conscious rats**

**
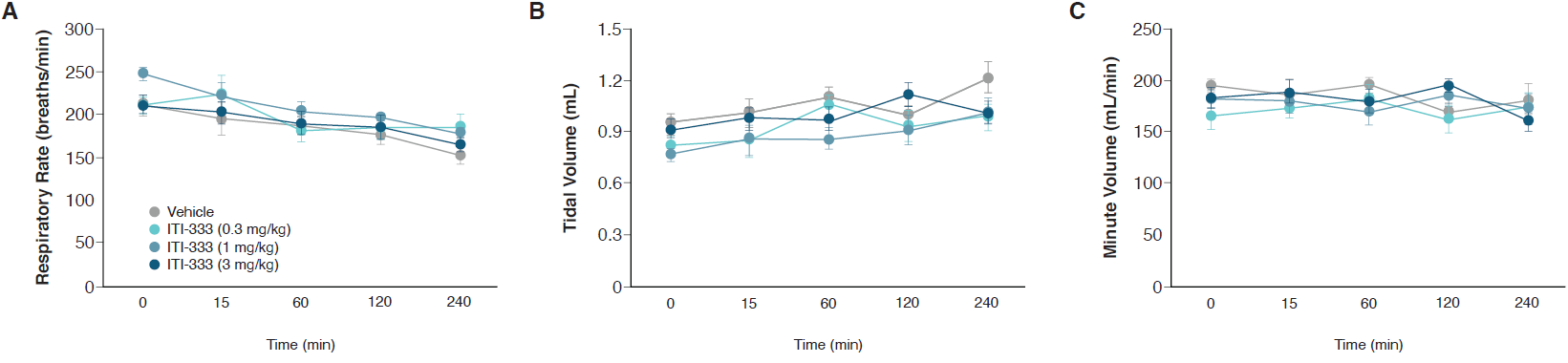
**

a) Respiratory rate, b) tidal volume, and c) minute volume were similar for rats treated with ITI-333 (0.3, 1, or 3 mg/kg) and rats treated with vehicle
